# News and Commercials: Binding Deficits for Complex Information in Schizophrenia

**DOI:** 10.3389/fpsyt.2020.611176

**Published:** 2021-01-20

**Authors:** Karolina Sejunaite, Claudia Lanza, Frederic Gaucher, Roland Klug, Matthias W. Riepe

**Affiliations:** Division of Geriatrics and Old Age Psychiatry, Psychiatry II Ulm University, Ulm, Germany

**Keywords:** recognition, memory binding, episodic memory, false memories, daily life

## Abstract

Memory for complex content is severely impaired in patients with schizophrenia spectrum disorders, which might make processing of daily information such as news and commercials particularly challenging. The goal of the present study was to assess the impairment of everyday memory in patients with schizophrenia. Healthy controls (HC) and patients with schizophrenia (SZ) were asked to watch a selection of six news segments and six commercials and complete a recognition task on the content of these video clips. All participants completed a neuropsychological test battery comprising measures of attention, working and episodic memory, and executive function. The total number of correctly recognized items was significantly lower in the SZ group. In contrast, the number of false recognitions was alike in both news and commercials paradigm. We conclude that memory in patients with schizophrenia is more prone to omissions than distortions for complex everyday stimuli. The results offer further support for impaired binding in SZ patients. Memory in SZ suffices to reject false multi-feature items on grounds of identifying at least one feature as incorrect but does not suffice to recall all features of a complex item and affirm it as correct.

## Introduction

Memory for context-rich information is at the very core of our everyday life. Ideally, this information should be stored and retrieved in its original form; however, distortions are inseparable part of memory's normal functioning (1). Due to the nature of their disorder, discerning external information input from the one internally generated represents an additional challenge for patients with schizophrenia. Delusions characterized by fixed false thoughts and fixed false beliefs are one of the core symptoms of schizophrenia. At least some of these false beliefs can result from the inability to identify self-generated thoughts as such and to misattribute them to an external source (2). To what extent these symptoms contribute to false memory formation in schizophrenia and whether other cognitive deficits influence memory functioning is not yet clearly understood.

In patients with schizophrenia the distinction between internal verbal thoughts and perception of external conditions may be blurred and result in the misrepresentations of the original context of events thus leading to false memories (3). Similarly, the increase in false memory content has also been reported for information of varying affective valence (4). Difficulties in source monitoring are thought to contribute significantly to erroneous memory content (5, 6). However, these findings might not be universally applicable to all patients with schizophrenia. It was reported that while all schizophrenia patients have fewer correct recognitions compared to healthy controls, acutely delusional patients have twice as many false recognitions as healthy controls and schizophrenia patients without current delusions (7). Proneness to delusions (7) and positive schizotypy (8, 9) has been linked to false memories even in non-clinical populations. Similarly, decreased hit rates in recognition task, has been associated with negative schizophrenia symptoms (10).

Reduced memory function is indeed constantly documented as one of the largest cognitive impairments in schizophrenia that is independent of such factors as treatment, symptoms, duration and stage of the illness (11). These deficits have been shown for both working and episodic memory as well as both verbal and visual memory (12). Some studies reported that patients with schizophrenia are particularly impaired in the processing of context-rich information (13–17) and the ability to bind information into a coherent memory trace (12, 17).

Straightforward laboratory paradigms such as Deese-Roediger-McDermott (DRM) procedure (18) have dominated false memory research in the past decade (19). However, it has been argued that errors in such tasks do not fully account for memory failures in the real world (20) and do not sufficiently predict memory outcomes in real-life situations (21). Patients with schizophrenia tend to have abnormal patterns of semantic processing (22, 23), therefore memory tasks such as DRM may introduce a bias when generalizing their results to a wider spectrum of memory function in schizophrenia.

To summarize, memory deficits in general and false memories in particular seem to be a prominent feature of cognitive deficits in patients with schizophrenia. Despite this deficit already being reported in laboratory tasks, a more complex paradigm is needed to investigate the scope of these deficits in complex, ecologically valid tasks. Given the frequency and influence of news and commercials on decision-making and other cognitive processes, the present study aims to investigate recognition memory for news and commercials video clips. We hypothesized a significant difference in the number of memory errors between patients with schizophrenia and healthy controls as well as the association between memory errors and general psychopathology and other cognitive deficits.

## Methods

The present study was designed as an open-label, non-randomized, cross-sectional, mono-centric study. The study received approval of the local ethics committee of the Ulm University (233/15) and was performed in accordance with the local ethical standards of the Ulm University and the guidelines outlined in the declaration of Helsinki (24). All persons gave their informed consent prior to their participation in the study.

### Participants

Twenty stabilized inpatients with schizophrenia (SZ; 6 females, age 19–30 years) and 24 healthy controls [HC; 14 females; age 21–35 years; the results of this control group have also been published previously (25)] participated in the study. HC consisted of healthy volunteers recruited by local advertising. SZ were recruited from a special detection and treatment service for young adults with schizophrenic disorders. CNS and affective disorders as well as intellectual disability and addictive behavior were ruled out in all participants by taking medical history. The exclusion criterion for HC group was a MADRS score above 8. In SZ patients concurrent depressive mood or a reduced drive are frequent and bring about higher scores in the MADRS.

### Psychological Assessments

#### Clinical Scales

*Montgomery-Asberg Depression-Rating-Scale (MADRS)* (26, 27): The score in the MADRS reflects the affective state of the examinee as assessed by a health care professional and consists of 10 aspects to be evaluated: apparent sadness, communicated sadness, inner tension, sleep, appetite, concentration, impetus, callousness, pessimistic thoughts, and suicidal ideation. Each of the aspects is given a score from 0 to 6 according to its severity. The total score ranges from 0 to 60. Scores 0–8 indicate no depression, 8–16 a mild, 16–24 a moderate, and 24 and higher a severe depression.

*Positive and Negative Syndrome Scale (PANSS)* (28, 29): The score in the PANSS is suited for typological and dimensional assessment. It comprises 7 items targeting positive symptoms, 7 items targeting negative symptoms, and 16 items targeting general psychopathology. PANSS total score ranges from 30 to 210. In the original publication (28) patients with schizophrenia scored on average 18 points on positive scale, 21 points on negative scale and almost 38 on the general psychopathology scale.

#### Neuropsychological Tests

*Vocabulary Test (Wortschatztest; WST)* (30): The WST is a measure of crystallized intelligence and requires an examinee to find an actual word among five non-word distractors. The target words increase in difficulty as the test progresses. The number of correct answers (maximum 40) is counted and the raw value is converted into IQ scores. *California Verbal Learning Test (CVLT)* (31): The CVLT is a verbal memory test, assessing variables such as immediate recall, free, and cued recall after short delay, free, and cued recall after long delay as well as recognition. A list of 16 words (four words of each category: fruit, clothing, drinks, tools) is read to the participant a total of 5 times. After each round, the participant is encouraged to recall as many words as possible. Immediate recall is followed by a recall after 5 and 20 min intervals, respectively, and a Yes/No recognition task. CVLT parameters used in the analysis were total words recalled in initial trial (CVLT 1), last trial (CVLT 5), total words recalled through all trials (CVLT total), recall after 5 min (CVLT short), recall after 20 min (CVLT long), as well as hits and false positives in the recognition.

*Digit and Visual Span (Wechsler Memory Scale Revised, WMS-R)* (32): The Digit Span test comprises digit span forward and digit span backward. In the digit span forward the participants are asked to repeat a sequence of digits until either the maximum number of 8 digits per sequence is reached or until two consecutive incorrectly repeated sequences of the same length. In the digit span backwards condition the same procedure is applied with the task to repeat the digits backward. The same principle was implemented for the Visual Span using Corsi-block forward and backward. One point is given for each correct answer with scores ranging 0–12 except for the forward visual span, where scores range 0–14.

*Trail Making Tests A & B (TMT-A & TMT-B)* (33): The TMT are tests to assess visual attention and mental flexibility and requires an examinee to draw pencil lines in ascending order from 1 to 25 (TMT-A) and 25 encircled numbers and corresponding letters in an alternating order (TMT-B) that are randomly dispersed on a DIN-A-4 sheet. The discrepancy between the TMT-A and TMT-B (i.e., TMT-B minus TMT-A) is an indicator of deficits in mental flexibility. The instructions require working as fast as possible while maintaining maximum accuracy.

*Fluency Tasks (Regensburg Verbal Fluency Test; RWT)* (34): RWT assesses semantic and phonetic verbal fluency. An examinee is instructed to generate as many words as possible in 1 min that belong to the category “animals” (semantic verbal fluency) as well as words starting with the letters “P” and “S” (phonemic fluency).

### Experimental Paradigms

The experimental paradigm has been used previously (25, 35, 36) as a memory task representing the daily memory processes. To assess the relevance of this task all participants were asked whether they regularly watch news and commercials. Our results show that 67 and 53% of HC and SZ, respectively, regularly watch news. Likewise 33% of HC and 42% of SZ reported to watch commercials. Despite participants reporting that they less frequently watch commercials on purpose, we still consider this to belong to everyday life since participants are exposed to commercials on a daily basis in diverse settings (supermarket, TV, public transport, and so forth).

#### News

Six news videos were shown to the participants with each video lasting between 27 and 39 s. All videos were selected from the same German news agency (ARD) and were originally broadcasted between the 1980s and early 1990s. The news topics pertained to domestic affairs and had the same format with a speaker (3 female and 3 male speakers) and extra information such as a photo or a map being shown in the background. None of the news segments reported about an event with an ongoing affective relevance and none have been repeatedly discussed in the media in the recent years, therefore it can be assumed that news had no personal relevance to the participants.

#### Commercials

Six commercials were shown to the participants with each video being between 25 and 34 s. All clips were selected from the internet and were originally broadcasted between the 1990s and early 2000s. The content of the commercials pertained grocery shopping for brands that can still be found in the market today.

#### Memory Assessment

A questionnaire with 12 questions was designed for each video to assess the amount of correctly retrieved facts from the respective video. Six out of 12 questions could be answered from the information presented in the video whereas the remaining six questions asked about details that were made up by the investigators to assess the number of erroneous memory items. All questions were in a form of a statement and needed to be answered giving one of the three possible answer choices: “Yes” (the statement is true), “No” (the statement is false), and “Unknown” (the information has not been addressed or shown in the video). For example in the video on the license fees it was asked whether the news host wore glasses (“No”) and whether the barrette of the news host was of the same color as her necklace (“unknown”) and whether the politician arguing for a fee increase in wore a beard (“Yes”). As a further example in the video on the detergent commercial, it was asked whether the both women wore sunglasses (“Yes”), whether the cocktail glass was decorated with an umbrella (“Unknown”), and whether the sunshades were dotted (“No”). The total number of correct “yes” and “no” responses was counted to give the total score of true positives. Questions for which the correct answer would have been “unknown” but for which the participant answered with “yes” or “no” were counted as the total score of false positives.

At the end of the experimental part participants were asked to evaluate subjective feeling of difficulty of the questions on a 5-point Likert scale with “1” being very easy and “5” being very difficult as well as to give a subjective estimate of how many questions they answered correctly and how many questions were non-answerable (i.e., misleading/false items).

### Data Analysis

Statistical analysis was carried out using SPSS software (SPSS 25.0 for Windows, Armonk, NY, 2017). The normality of distribution was assessed by Shapiro–Wilk's test and additional visual inspection of the histograms. Median and interquartile (IQR) range are reported as measures of central tendency and dispersion. Due to small sample size, all group comparisons were calculated using the Mann–Whitney-*U*-test. A regression analysis was performed to identify the contribution of the psychopathological and cognitive factors to the memory function. Raw scores of hits and false alarms were used to calculate the discriminability (d′) and bias (C) according to Signal Detection theory (37). Effect sizes were calculated using Cohen's d. Performance on news and commercials memory task was analyzed separately due to differences in the density of factual information, rate of scene change, valence and musical background.

## Results

Demographic variables are shown in Table 1. The vocabulary test shows a difference in the raw scores (Table 1), however IQ scores for both groups were within a normal range (IQ HC: median: 101–133; IQ SZ: IQ 82–122). HC are slightly older than SZ. The median score for the SZ group lies within boundaries for moderate depression, with some SZ patients presenting with higher scores in the MADRS. In order to characterize the symptoms of SZ patients a PANSS was performed. Median scores for positive symptoms [Median (Mdn) = 18, interquartile range (IQR) = 14–20], negative symptoms [Mdn = 14, IQR = 13–16] and general psychopathology [Mdn = 33, IQR = 28–34] are in good harmony with the typical scores of SZ patients (28).

**Table 1:** Demographic variables of study participants.

	**HC (*n* = 24)**	**SZ (*n* = 20)**	***U***	***Z***	***p***	**Cohen's d**
	**Median (IQR)**	**Median (IQR)**				
Age	26.5 (24–29)	23.5 (20–28)	142.5	−2.3	0.021	0.75
WST	35.0 (33–37)	31.0 (25–33)	53.5	−4.2	<0.001	1.48
Education (years)	13.0 (13–17)	10.0 (10–12)	47.5	−4.9	<0.001	1.98
IQ	114.0 (108–122)	101.0 (89–106)	45.5	−4.7	<0.001	1.58
MADRS	0 (0–1)	21.0 (14–29)	8.0	−5.6	<0.001	2.70

*HC, Healthy controls; IQ, Intelligence quotient; IQR, Interquartile range; MADRS, Montgomery-Åsberg Depression Rating Scale; SZ, Patients with schizophrenia; WST, Wortschatzstest*.

The results of neuropsychological assessment are shown in Table 2. HC outperformed SZ in all tests representing attention, working memory, cognitive flexibility, and verbal memory.

**Table 2:** Results of neuropsychological testing.

	**HC (*n* = 24)**	**SZ (*n* = 20)**	***U***	***Z***	***p***	**Cohen's d**
	**Median (IQR)**	**Median (IQR)**				
DS forward	10 (9–11)	8.5 (6–10)	138.5	−2.4	0.015	0.88
DS backward	9 (7–11)	5.5 (4–8)	96	−3.4	0.001	1.21
VS forward	10 (9–12)	8 (6–10)	108	−3.1	0.002	1.02
VS backward	10 (8–11)	8 (6–10)	150	−2.2	0.031	0.70
TMT-A	19.5 (16–25)	30.5 (21–38)	94.5	−3.4	0.001	1.18
TMT-B	41.5 (35–63)	67.5 (55–92)	81.5	−3.7	<0.001	1.12
TMT B–A	21.5 (16–42)	39 (31–65)	133.5	−2.5	0.012	0.87
Semantic fluency	23 (21–26)	17.5 (14–20)	68	−4.1	0.001	1.26
Phonemic fluency P	12 (9–16)	9 (6–11)	125	−2.7	0.006	0.93
Phonemic fluency S	15 (12–20)	12 (9–15)	111	−3.1	0.002	1.00
CVLT 1	7 (6–8)	4.5 (3–6)	81.5	−3.8	<0.001	1.34
CVLT 5	16 (14–16)	10 (9–13)	44	−4.7	<0.001	2.04
CVLT total	65 (55–67)	39 (34–51)	34	−4.9	<.001	2.04
CVLT short	16 (15–16)	12 (9–14)	70	−4.1	<0.001	1.80
CVLT long	16 (14–16)	11 (9–14)	74.5	−4.0	<0.001	1.58
CVLT hits	16 (16–16)	16 (15–16)	159	−2.5	0.014	1.95
CVLT FP	0 (0–0)	0 (0–2)	153.5	−2.9	0.004	0.33

*CVLT, California Verbal Learning Test; DS, digit span; FP, false positives; HC, healthy controls; SZ, Patients with Schizophrenia; TMT, Trail Making Test; VS, visual span*.

In the memory assessment for news and commercials there was a difference in the number of hits for both news and commercials, whereas the number of false alarms was alike in both conditions. There was a difference in discriminability for both news and commercials with HC group being significantly better in discriminating between hits and false alarms. There was also a difference in answering strategies between the two groups. HC displayed a liberal answering tendency, classifying recognition items as previously encountered in the video, whereas the SZ group leaned toward a conservative answering tendency classifying recognition items as novel for the most trials. There were no group differences in subjective difficulty. When asked to estimate their performance, patients with SZ gave a lower subjective estimate for hits compared to the HC group; however, the subjective estimate of correctly rejected false items did not differ between the groups (Table 3).

**Table 3:** Experimental paradigm results.

	**HC (*n* = 24)**	**SZ (*n* = 20)**	***U***	***Z***	***p***	**Cohen's d**
	**Median (IQR)**	**Median (IQR)**				
**News**						
Hits	28.5 (27/30)	22 (19–25)	26.5	−5.1	<0.001	2.34
FP	11 (9/14)	11.5 (7–20)	227.5	−0.29	0.768	0.37
d′	1.2 (1/1.5)	0.65 (0.07/1.03)	93.5	−3.5	0.001	1.15
c	−0.1 (−0.33/−0.01)	0.05 (−0.25/0.36)	143	−2.3	0.022	0.58
Subjective difficulty	3 (3/4)	3 (3/4)	198	−0.04	0.965	0.06
Estimated hits	45.5 (35/50)	35 (25/40)	78.5	−1.9	0.046	0.74
Estimated false items	20 (15/30)	16 (10/20)	97	−1.3	0.186	0.39
**Commercials**						
Hits	30 (28–32)	23.5 (19–26)	40.5	−4.4	<0.001	1.91
FP	10 (7–13)	10.5 (6–17)	191.5	−0.48	0.628	0.30
d′	1.5 (1.26/1.74)	1.1 (0.29/1.34)	32	−2.8	0.004	1.24
c	−0.15 (−0.39/0.03)	0.13 (−0.24/0.34)	47.5	−1.8	0.075	0.87
Subjective difficulty	3 (2–3)	3 (2–4)	184.5	−0.43	0.669	0.27
Estimated hits	49 (43–56)	40 (30–50)	84	−1.8	0.073	0.76
Estimated false items	21 (15–26)	15 (10–25)	89	−1.6	0.107	0.57

*c, response bias; d′, discriminability; HC, healthy controls; SZ, Patients with Schizophrenia*.

**Figure 1:**
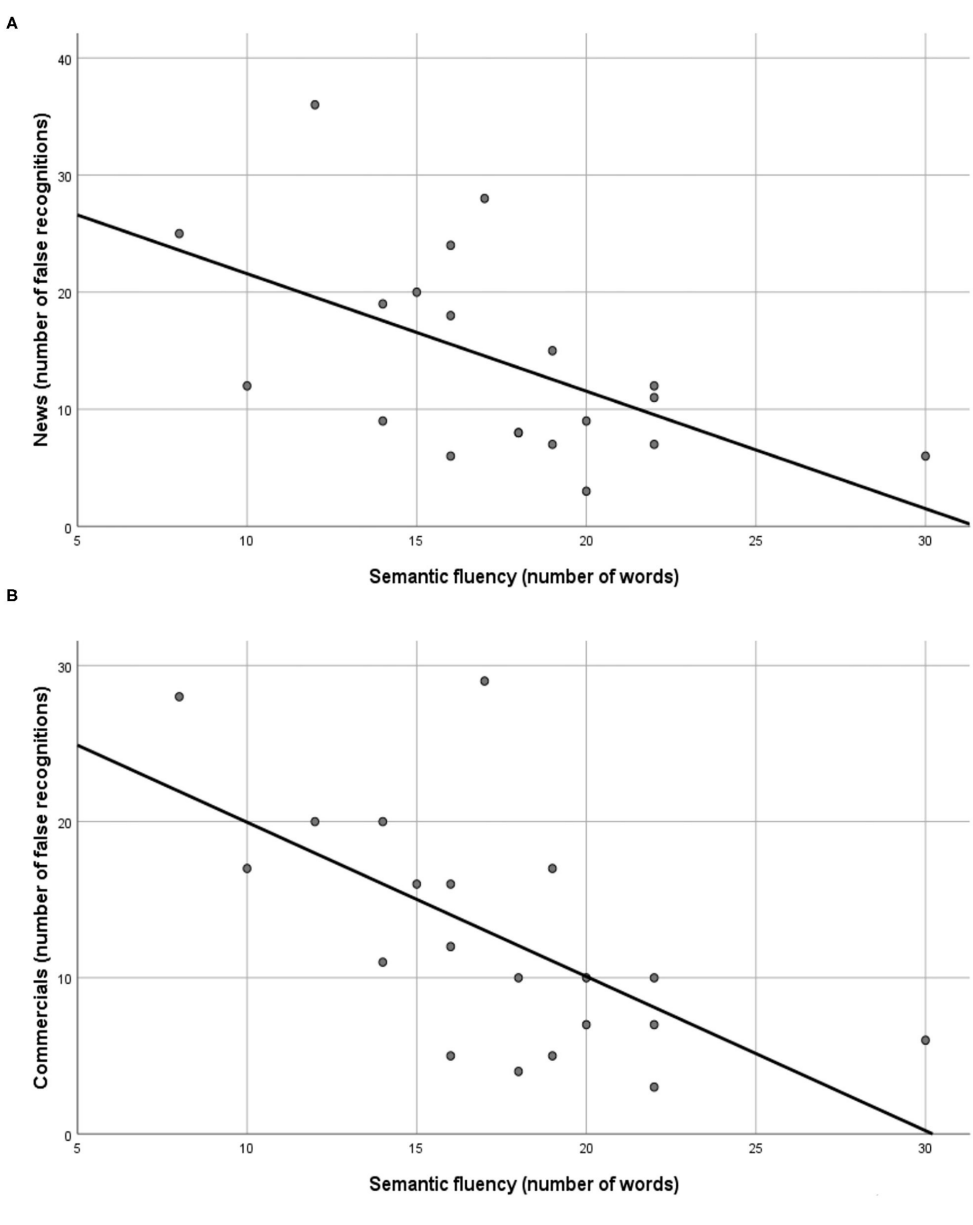
Association between false positive answers and semantic fluency in the **(A)** news and **(B)** commercials paradigms.

To explain variables contributing to the variance in the rate of false positive answers for the experimental paradigm within patients with SZ, a stepwise regression analysis per performed. The model included PANSS negative symptoms, PANSS positive symptoms, PANSS general psychopathological score, MADRS, age, difference between TMT-B and TMT-A, and semantic fluency. Results revealed a strong association of the number of false positives with semantic fluency for both, the news and the commercials paradigm (Table 4). In both cases, the number of false positives increased as the performance on semantic fluency decreased (Figure 1). In both cases semantic fluency explained between 30 and 40% of the variance in false positive answers. The model for false positive answers in the news paradigm additionally included the MADRS score. Interestingly, it seems that false positive answers among patients with SZ increase as their MADRS score decreases.

**Table 4:** Regression analysis of false positive answers in the news and commercials paradigms.

	***R^**2**^***	**Adj. *R^**2**^***	**Regression coefficient B**	**Standard error**	**Standardized coefficient Beta**	***t***	***p***
**News false positives**							
Model^*^							
Semantic fluency	0.553	0.305	−1.026	0.304	−0.570	−3.375	0.004
MADRS	0.738	0.545	−0.399	0.137	−0.490	−2.901	0.010
**Commercials false positives**							
Model^**^							
Semantic fluency	0.637	0.406	−0.994	0.292	−0.637	−3.408	0.003

* *Model including PANSS negative symptoms, PANSS positive symptoms, PANSS general psychopathological score, MADRS, age, difference between TMT-B and TMT-A, and semantic fluency*.

** *Model including PANSS negative symptoms, PANSS positive symptoms, PANSS general psychopathological score, MADRS, age, difference between TMT-B and TMT-A, and semantic fluency*.

*MADRS, Montgomery-Asberg-Depression-Rating-Scale; PANSS, Positive and Negative Syndrome Scale; TMT, Trail Making Test*.

## Discussion

The present study employed a task using ecologically valid stimuli comprising news and commercial videos. The aim of the study was to assess recognition memory for such daily encountered material among healthy controls (HC) and patients with schizophrenia (SZ).

Contrary to our expectations, there was no increase in the rate of false positives in SZ, although HC outperformed SZ in the number of hits by a significant margin. In good harmony with this result, HC group showed significantly better discriminability, which boosted their hit rate. Answering strategies also differed significantly between the groups. SZ group contrary to the HC preferred a conservative answering strategy, meaning their threshold for classifying an item as previously encountered was higher, thus allowing them to avoid false alarms, which might have occurred due to their lower discriminability. Although patients with SZ showed inferior performance compared to HC in a multitude of neuropsychological domains such as attention, executive functions, and episodic memory, only semantic fluency seemed to be inversely and significantly associated with false memories and was able to explain between 30 and 40% of variance in this variable.

Comparable decrease in recognition hits by simultaneously good ability to correctly reject false items in recognition tasks was already demonstrated among patients with schizophrenia using emotionally charged and delusion-related videos (38). The rate of false positive answers at the control level was also found in another study, but only among patients experiencing no delusions at that point of time (7). However, contrary to our expectation, no association of false recognitions with positive or negative subscales of the PANSS or the general psychopathology were found in the current study. Possibly, the lack of association results from the absence of severe symptoms, especially severe positive symptomatology, as most of the patients were already past the initial acute phase. The pattern found in the present and the previously mentioned study might help to differentiate between memory profiles of patients in different stages of the disease.

Within the group of patients with SZ lower performance in semantic fluency task was associated with higher number of false positive answers in the recognition task. This finding is in good harmony with the previously demonstrated association between false memory rates and reduced processing of semantic relationships as an indicator of an inability to implement explicit relational processing strategies (39). Likewise, schizophrenia has been found to be associated with a failure to integrate contextual semantic cues to generate a multi-dimensional memory unit (17). This integration of contextual cues, i.e., binding, depends on processes active while encoding the information (40). Binding deficits have been repeatedly reported in numerous studies (13, 17, 41, 42). Inability to bind features in order to create a complex representation is likely to occur in early stages of encoding (13, 43). This might explain the decreased rate of hits and seemingly intact ability to reject false items in recognition. Since a complex item needs to be either rejected or accepted as a whole, it is likely that a complex item is easier to reject when patients are sure that at least one of the components of such an item is false. In contrast, all items of a correct complex statement should be recognized for an item to be confidently accepted as previously encountered.

There are several limitations to the study. Firstly, there was a group difference in mood, which seemed to contribute to the rate of false positive answers among patients with SZ in a counterintuitive manner. This relationship between mood and false memory in schizophrenia needs to be addressed in a more systematic way in future studies. Furthermore, the influence of antipsychotic medications could not be accounted for, as the data on exact dosage and duration of treatment was not available for all participants in the SZ group.

## Conclusion

Schizophrenia patients suffer from deficits in multiple cognitive areas, memory being most pronounced. Using an ecologically valid recognition paradigm, the current study was able to demonstrate that whereas the hit rate is significantly decreased among young patients with schizophrenia, the rate of false positive answers is at the control level. This heterogeneous memory pattern is likely caused by binding deficits, which make multi-feature information harder to encode correctly, while simultaneously making it easier to reject the information, if it is false.

## Data Availability Statement

The raw data supporting the conclusions of this article will be made available by the authors, without undue reservation.

## Ethics Statement

The studies involving human participants were reviewed and approved by Ethikkommission der Universität Ulm. The patients/participants provided their written informed consent to participate in this study.

## Author Contributions

KS, CL, and FG were involved in acquisition of the data, data analysis, and drafting, and revising the manuscript. KS, CL, RK, and MR were involved in designing the study, interpretation of the data, and drafting and revising the manuscript. All authors approved the final version of the manuscript.

## Conflict of Interest

The authors declare that the research was conducted in the absence of any commercial or financial relationships that could be construed as a potential conflict of interest.
